# Assessment of body composition and prediction of infectious pancreatic necrosis via non-contrast CT radiomics and deep learning

**DOI:** 10.3389/fmicb.2024.1509915

**Published:** 2024-12-13

**Authors:** Bingyao Huang, Yi Gao, Lina Wu

**Affiliations:** ^1^Department of Radiology, Shengjing Hospital of China Medical University, Shenyang, China; ^2^Department of Laboratory Medicine, Shengjing Hospital of China Medical University, Shenyang, China

**Keywords:** pancreatitis, infectious pancreatic necrosis, artificial intelligence, adipose tissue, musculature

## Abstract

**Aim:**

The current study aims to delineate subcutaneous adipose tissue (SAT), visceral adipose tissue (VAT), the sacrospinalis muscle, and all abdominal musculature at the L3–L5 vertebral level from non-contrast computed tomography (CT) imagery using deep learning algorithms. Subsequently, radiomic features are collected from these segmented images and subjected to medical interpretation.

**Materials and methods:**

This retrospective analysis includes a cohort of 315 patients diagnosed with acute necrotizing pancreatitis (ANP) who had undergone comprehensive whole-abdomen CT scans. The no new net (nnU-Net) architecture was adopted for the imagery segmentation, while Python scripts were employed to derive radiomic features from the segmented non-contrast CT images. In light of the intrinsic medical relevance of specific features, two categories were selected for analysis: first-order statistics and morphological characteristics. A correlation analysis was conducted, and statistically significant features were subjected to medical scrutiny.

**Results:**

With respect to VAT, skewness (*p* = 0.004) and uniformity (*p* = 0.036) emerged as statistically significant; for SAT, significant features included skewness (*p* = 0.023), maximum two-dimensional (2D) diameter slice (*p* = 0.020), and maximum three-dimensional (3D) diameter (*p* = 0.044); for the abdominal muscles, statistically significant metrics were the interquartile range (IQR; *p* = 0.023), mean absolute deviation (*p* = 0.039), robust mean absolute deviation (*p* = 0.015), elongation (*p* = 0.025), sphericity (*p* = 0.010), and surface volume ratio (*p* = 0.014); and for the sacrospinalis muscle, significant indices comprised the IQR (*p* = 0.018), mean absolute deviation (*p* = 0.049), robust mean absolute deviation (*p* = 0.025), skewness (*p* = 0.008), maximum 2D diameter slice (*p* = 0.008), maximum 3D diameter (*p* = 0.005), sphericity (*p* = 0.011), and surface volume ratio (*p* = 0.005).

**Conclusion:**

Diminished localized deposition of VAT and SAT, homogeneity in the VAT and SAT density, augmented SAT volume, and a dispersed and heterogeneous distribution of abdominal muscle density are identified as risk factors for infectious pancreatic necrosis (IPN).

## 1 Introduction

Acute necrotizing pancreatitis (ANP), a grave complication of acute pancreatitis (AP), arises from the aberrant activation of pancreatic digestive enzymes, resulting in tissue necrosis. This necrotic tissue creates an environment conducive to bacterial proliferation, which frequently precipitates infectious pancreatic necrosis (IPN), occurring in approximately 30% of ANP patients and manifesting a mortality rate as high as 30%. Consequently, the early and precise diagnosis and treatment of IPN are of paramount importance. Given the intimate connection between the pathophysiology of ANP and systemic metabolism, recent research has focused on the roles of muscle and adipose tissue in ANP and its complications (Yee et al., 2021; Zhang X. et al., 2024).

In unraveling the intricate pathogenesis of IPN, it is essential to consider the potential contributions of body composition, particularly with respect to muscle and fat. Skeletal muscle, a fundamental component of the human body, not only underpins essential motor functions but is also intimately associated with an individual’s metabolic state, inflammatory responses, and long-term clinical outcomes. Adipose tissue, especially visceral fat, is recognized as a “metabolically active” entity that secretes various bioactive molecules involved in regulating energy metabolism, inflammatory reactions, and immune functions. Recent evidence suggests that alterations in muscle and fat content and distribution may exert direct or indirect influences on the progression and outcomes of AP (Fu et al., 2023; Dawra et al., 2023). Nonetheless, a comprehensive understanding of the specific roles these changes play in the development of IPN remains elusive. This study seeks to bridge this gap by examining the distinctive alterations in muscle and fat among IPN patients, thereby offering a novel perspective on the multifaceted disease trajectory of IPN. While the previous studies have acknowledged the significance of assessing body composition, they have been mainly confined to macroscopic observations and correlative analyses, with a limited exploration of the precise mechanisms by which muscle and fat impact IPN. Hence, this study will utilize CT scans to meticulously evaluate the distribution of muscle and fat in IPN patients. This methodological approach is anticipated to elucidate the intrinsic correlation between muscle and fat status and the pathophysiological underpinnings of IPN, providing a scientific foundation for early diagnosis, therapeutic strategy development, and prognostic enhancement. The objective is to furnish new theoretical insights and practical guidance for precision medicine and IPN management.

Computed tomography (CT), as a prevalent imaging technique, offers distinct advantages in assessing body composition. It not only delineates the distribution of muscles and fats with clarity but also quantitatively analyzes critical parameters, such as muscle area and fat content, through precise measurement tools, thereby affording clinicians a wealth of morphological and functional information (Zhang R. et al., 2023; Vogele et al., 2023). In the evaluation of pancreatitis, CT accurately portrays pancreatic morphological alterations, necrotic regions, and the spread of inflammation, playing a pivotal role in diagnosing ANP and monitoring disease progression (Balthazar et al., 1990). Moreover, CT has proven to be particularly adept at assessing muscle and fat, enabling the exact measurement of their distribution and proportions, which is instrumental in evaluating patients’ nutritional status, inflammatory responses, and disease prognoses (Hou et al., 2024). The advent of artificial intelligence technology, particularly the extensive application of deep learning algorithms in medical image processing, has introduced a novel perspective and set of tools for exploring the complex pathological mechanisms of AP (Zhang C. et al., 2024; Yin et al., 2024). By leveraging deep learning algorithms, we can uncover the profound features embedded within the vast repository of CT image data (Zhang R. et al., 2023), which may be intimately linked to the pathological changes of IPN, thereby facilitating early prediction and precise treatment of the condition. Despite the limitations of non-enhanced CT in the traditional visual diagnosis of pancreatic diseases due to the lack of contrast, its amalgamation with radiomics technology has yielded promising diagnostic outcomes (Koç and Taydaş, 2020; Janisch et al., 2022; Cao et al., 2023).

This study explores the utility of body composition assessment based on non-contrast CT in ANP patients and harnesses deep learning and radiomics techniques to delve into the potential connections between body components and the onset of IPN. Additionally, we aspire to provide novel insights and strategies for the early detection and personalized treatment of the disease by examining the interplay with body composition.

## 2 Materials and methods

### 2.1 Patients

**Figure 1:**
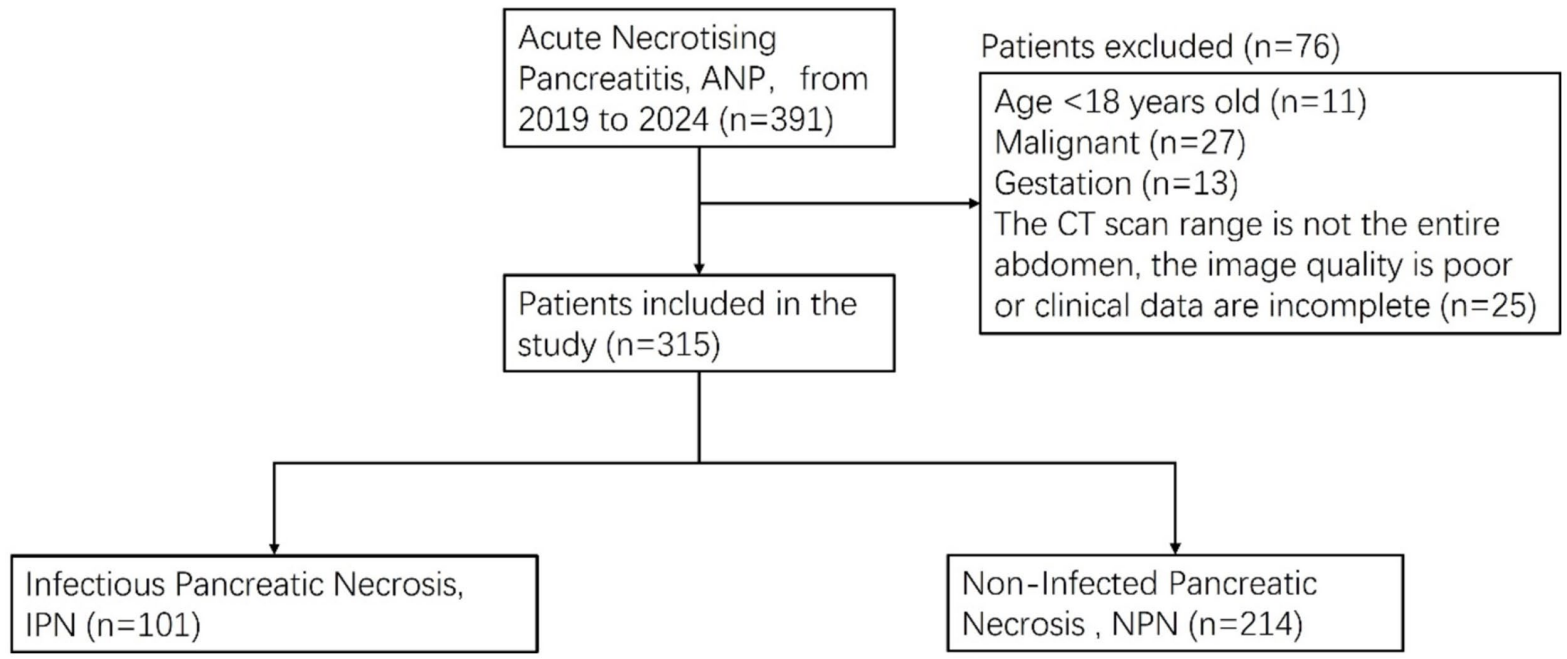
Patients’ enrollment and exclusion process in the infectious pancreatic necrosis (IPN) database.

This study was conducted in accordance with the Declaration of Helsinki and received ethical approval from the Ethics Committee of Shengjing Hospital at China Medical University, with a waiver of informed consent for participants (ethical approval number: 2024PS1480K). As depicted in Figure 1, we conducted a retrospective analysis of data from patients who were diagnosed with ANP and admitted to our institution between March 2019 and August 2024 and underwent CT scans within a week of symptom onset. Inclusion criteria included CT scans performed within 1 week of admission. Exclusion criteria included: (1) pregnancy; (2) age below 18 years; (3) concurrent malignancy; and (4) non-whole-abdomen CT scans, poor image quality, or incomplete clinical data that could compromise the accuracy and reliability of the assessment outcomes.

Clinical data collected included age, sex, IPN status, diabetes, hypertension, hyperlipidemia, hypoxemia, coronary heart disease, gallstone pancreatitis, mechanical ventilation, and hospital stay duration.

### 2.2 CT image acquisition

Patients were subjected to whole-abdomen CT imaging within 1 week following admission. All scans were performed with the patients in a supine position during inhalation using a (1) Philips Brilliance ICT 256-slice spiral CT scanner (Philips Healthcare). (2) Python package PyRadiomics version 3.0.1 (Python Software Foundation). (3) Statistical Package for the Social Sciences (SPSS) version 26.0 (IBM Corp). The scanning field extended from the diaphragmatic dome to the pubic symphysis. Scan parameters were set as follows: tube voltage at 120 kV, tube current adjusted to automatic milliamperage, matrix size of 512 × 512, a pitch of 1, with routine images at a slice thickness of 3.0 mm, and thin-section images at 1.0 mm intervals.

### 2.3 Data annotations

**Figure 2:**
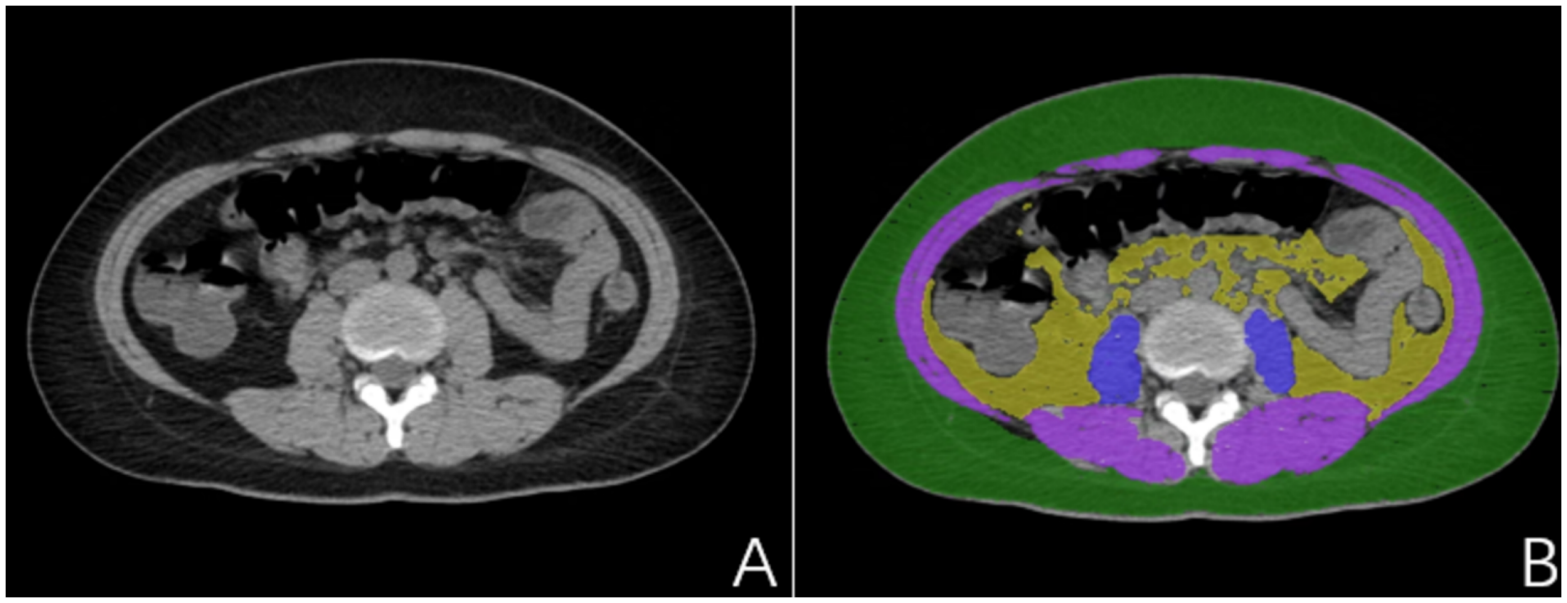
**(A)** Non-pancreatitis crowd image; **(B)** Delineated subcutaneous adipose tissue (SAT) (green), visceral adipose tissue (VAT) (yellow), sacrospinalis (blue), and abdominal muscles (blue and purple).

The present study utilized a stringent data annotation protocol to guarantee precision and consistency. To ensure the model’s generalizability and to reduce the interference of pancreatitis on the delineation of visceral fat, we employed CT images from a distinct cohort of individuals without pancreatitis for region of interest (ROI) annotation. Two experienced radiologists, each with over 5 years of expertise in diagnostic imaging and unaware of the study’s aims, initially demarcated the subcutaneous fat (SAT), visceral fat (VAT), sacrospinalis, and all abdominal muscles at the L1–S1 vertebral level (Figure 2). To further bolster the reliability of the data annotation, a seasoned diagnostic radiologist with over 15 years of experience, also unfamiliar with the study’s objectives, was brought in to scrutinize the ROIs. This senior radiologist, well-versed in medical imaging and rich in clinical diagnostic acumen, meticulously reviewed and corrected the annotations made by the junior physicians; during the review, the senior radiologist engaged in profound discussions with the junior physicians regarding controversial or unclear areas. However, these discussions were restricted to technical matters and excluded any discourse on the patient’s clinical conditions or the study’s hypotheses. Consensus was achieved through negotiation to ensure that each ROI annotation was exact and precise. This blinded data annotation process helped minimize the influence of subjective bias on the study’s outcomes.

### 2.4 Segmentation network

For the segmentation model, we adopted a 5-fold cross-validation approach with a data partition ratio of 5:1. Specifically, the dataset was initially randomly divided into five subsets, with four subsets (80%) serving as the training set and the remaining subset (20%) as the validation set. This procedure was replicated 5 times to ensure that each data point was used as a validation set exactly once. This ensures that we can maximize data utilization while more accurately evaluating the model’s generalization ability.

A neural network architecture was employed for accurate image segmentation. The process began with the segmentation of the L3–L5 vertebrae. For ROI regions with smaller initial segmented areas and errors, erosion processing was applied to enhance their accuracy. Following this, connected component analysis was conducted, revealing that the segmentation accuracy of the L4 vertebra was the highest. Using this information, we expanded one connected component upward and one downward to accurately identify the L3–L5 vertebral region. Subsequently, the muscles and fat within this region were delineated.

Our segmentation network was constructed based on the nnU-Net architecture (Isensee et al., 2021). nnUNet can automatically perform preprocessing based on the characteristics of the dataset. Additionally, it offers various architectures that handle 3D matrices effectively, making it highly suitable for CT images. Our segmentation network exclusively used non-enhanced CT images as a data source.

### 2.5 Evaluation of ANP and IPN

**Figure 3:**
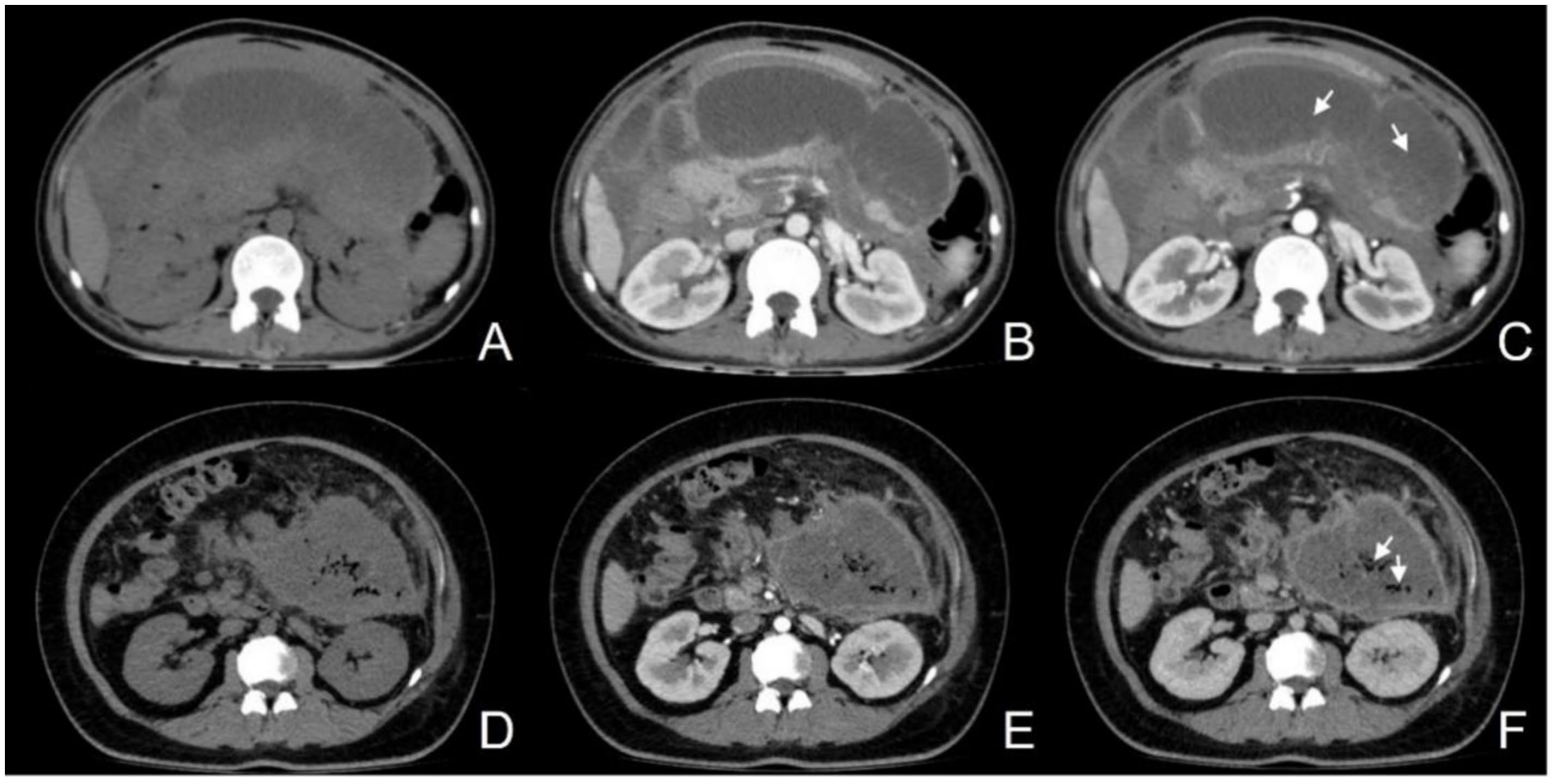
**(A–C)** Female, 50-year-old, acute necrotizing pancreatitis (ANP), showing a slightly hypodense lesion without enhancement after contrast (**C**, arrow); **(D–F)** Female, 31-year-old, infectious pancreatic necrosis (IPN), with scattered gas within the necrosis (**F**, arrow).

For the assessment of ANP (Figures 3A–C), all case evaluations were conducted by two radiologists specializing in imaging diagnostics, each with over 8 years of diagnostic experience, who reviewed all imaging studies performed during the inpatient stay of the enrolled patients, devoid of any clinical information and adverse outcomes. Regarding the assessment of IPN (Figures 3D–F), an abdominal CT specialist with over a decade of diagnostic experience evaluated the cases by synthesizing clinical data, imaging findings, and laboratory test results. It is pertinent to note that IPN was defined as the initial percutaneous catheter drainage or surgical retrieval, yielding a positive culture or the observation of extraluminal gas on CT scans. Pancreatic necrosis refers to areas within the pancreatic parenchyma that exhibit hypoattenuation or lack of enhancement on CT imaging. Peripancreatic necrosis is characterized by collections containing varying amounts of fluid and necrotic tissue associated with necrotizing pancreatitis, and it can be diagnosed when non-liquid components of non-enhancing areas are visualized on CT scans.

### 2.6 Radiomics feature selection

Utilizing the Python package PyRadiomics version 3.0.1, we conducted the extraction of radiomic features from non-contrast CT images that had been segmented automatically. In recognition of the intrinsic clinical relevance of specific attributes, we narrowed our focus to two principal categories: first-order and shape characteristics. The first-order features encapsulate the distribution patterns of pixel intensities within the images (refer to Table 1, items 1–18), whereas the shape features concentrate on delineating the geometric attributes of the imaged structures (refer to Table 1, items 19–32).

**Table 1:** Radiomics features and their mathematical explanations used in this study.

Number	Feature	Mathematical explanation
1	10 percentile	The 10th percentile value of the voxel intensities within the region of interest (ROI). This is the value below which 10% of the data falls.
2	90 percentile	The 90th percentile value of the voxel intensities within the ROI. This is the value below which 90% of the data falls.
3	Energy	A measure of the magnitude of voxel values in an image. It is the sum of the squares of the voxel values.
4	Entropy	Specifies the uncertainty/randomness in the image values. It measures the average amount of information required to encode the image values.
5	IQR	The difference between the 75th percentile (Q3) and the 25th percentile (Q1) of the voxel intensities within the ROI. It is a measure of the spread of the middle 50% of the data.
6	Kurtosis	A measure of the “peakedness” of the distribution of voxel intensities within the ROI. A high kurtosis value indicates a sharp peak and heavy tails, while a low kurtosis value indicates a flat distribution.
7	Maximum	The maximum voxel intensity value within the ROI.
8	Mean	The average voxel intensity value within the ROI.
9	MAD	The average of the absolute differences between the individual voxel intensities and the mean voxel intensity.
10	Median	The middle value of the voxel intensities within the ROI, such that half of the data is above and half is below this value.
11	Minimum	The minimum voxel intensity value within the ROI.
12	Range	The difference between the maximum and minimum voxel intensity values within the ROI.
13	Robust MAD	A measure of the spread of the data that is less sensitive to outliers than the MAD.
14	Root mean squared	The square root of the mean of the squares of the differences between the individual voxel intensities and the mean voxel intensity.
15	Skewness	A measure of the asymmetry of the distribution of voxel intensities within the ROI. A positive skewness indicates a tail on the right side of the distribution, while a negative skewness indicates a tail on the left side.
16	Total energy	The energy feature scaled by the volume of the voxel in cubic mm. It takes into account both the magnitude of the voxel values and the size of the ROI.
17	Uniformity	A measure of the homogeneity of the voxel intensities within the ROI. It is the sum of the squares of each voxel intensity value divided by the square of the sum of the voxel intensity values.
18	Variance	The average of the squared differences between the individual voxel intensities and the mean voxel intensity. It measures the spread of the data around the mean.
19	Elongation	The elongation of the ROI shape is a measure of the relationship between the two largest principal components of the ROI.
20	Flatness	The flatness of the ROI shape is a measure of the relationship between the largest and smallest principal components of the ROI.
21	Least axis length	The length of the smallest principal axis of the ROI.
22	Major axis length	The length of the largest principal axis of the ROI.
23	Maximum 2D diameter column	The maximum 2D diameter of the ROI in the column direction (typically the *y*-axis in an image).
24	Maximum 2D diameter row	The maximum 2D diameter of the ROI in the row direction (typically the *x*-axis in an image).
25	Maximum 2D diameter slice	The maximum 2D diameter of the ROI in the slice direction (typically the *z*-axis in a 3D image).
26	Maximum 3D diameter	The maximum 3D diameter of the ROI is the largest Euclidean distance between any two points on the surface of the ROI.
27	Mesh volume	The volume of the ROI is calculated from the triangular mesh that represents the surface of the ROI.
28	Minor axis length	The length of the second-largest principal axis of the ROI
29	Sphericity	A measure of how spherical the ROI is. It is the ratio of the surface area of a sphere with the same volume as the ROI to the actual surface area of the ROI.
30	Surface area	The surface area of the ROI.
31	Surface volume ratio	The ratio of the surface area of the ROI to its volume. A lower value indicates a more compact, spherical shape.
32	Voxel volume	The volume of a single voxel within the ROI.

### 2.7 Statistical analysis

Statistical computations were executed utilizing Statistical Package for the Social Sciences (SPSS) version 26.0. Quantitative data were expressed in terms of mean deviation $\left(\bar{x}\pm s\right)$ and were subjected to comparison via *t*-tests. Qualitative data were represented in frequencies and were evaluated using the *χ*^2^/Fisher exact tests, as appropriate. In radiomic data, the *t*-tests were employed for data that exhibited a normal distribution. In contrast, the Mann–Whitney *U* test was utilized for data that did not conform to a normal distribution. *p* < 0.05 was deemed indicative of statistical significance.

**Figure 4:**
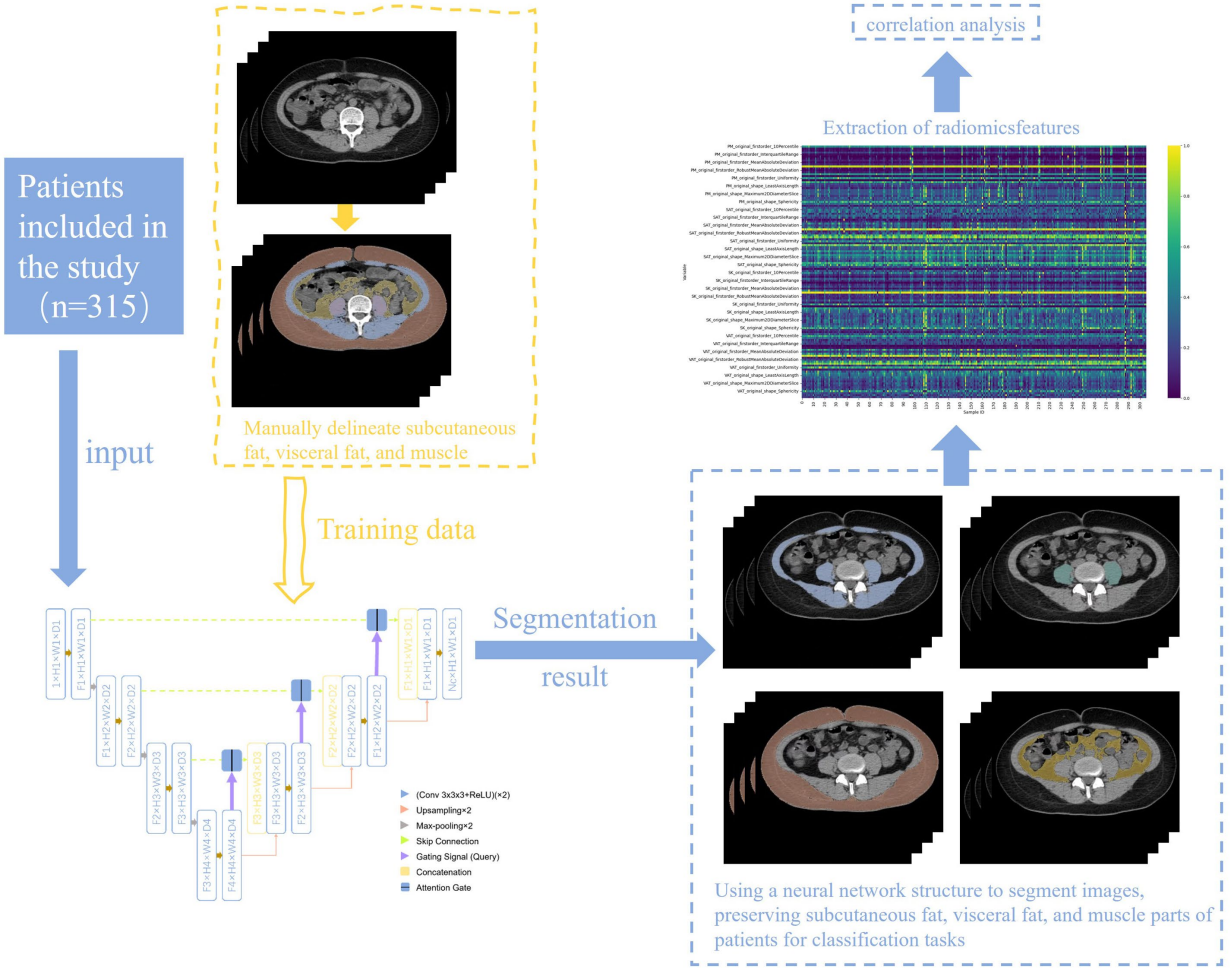
Study flowchart.

The overall study workflow is shown in Figure 4.

## 3 Results

### 3.1 Clinical characteristics

The present study enrolled a total of 315 patients. Table 2 delineates the clinical attributes of the participants within the IPN and non-infected pancreatic necrosis (NPN) cohorts.

**Table 2:** Comparison of clinical characteristics between infectious pancreatic necrosis (IPN) and non-infected pancreatic necrosis (NPN) groups.

Clinical characteristics		IPN (*n* = 101)	NPN (*n* = 214)	*p*
Sex (*n*)	Male	70 (69.3%)	143 (66.8%)	0.66
	Female	31 (30.7%)	71 (33.2%)	
Age (years)		47.91 ± 15.45	44.14 ± 13.85	0.038
Diabetes mellitus (*n*)	Yes	44 (43.6%)	98 (45.8%)	0.71
	No	57 (56.4%)	116 (54.2%)	
Hypertension (*n*)	Yes	35 (34.7%)	61 (28.5%)	0.269
	No	66 (65.3%)	153 (71.5%)	
Hyperlipidemia (*n*)	Yes	44 (43.6%)	108 (50.5%)	0.252
	No	57 (56.4%)	106 (49.5%)	
Coronary heart disease (n)	Yes	5 (5.0%)	8 (3.7%)	0.762
	No	96 (95.0%)	206 (96.3%)	
Mechanical ventilation (*n*)	Yes	32 (31.7%)	19 (8.9%)	<0.001
	No	69 (68.3%)	195 (91.1%)	
Biliary pancreatitis (*n*)	Yes	13 (12.9%)	26 (12.1%)	0.856
	No	88 (87.1%)	188 (87.9%)	
Concurrent hypoxemia (*n*)	Yes	31 (30.7%)	49 (22.9%)	0.138
	No	70 (69.3%)	165 (77.1%)	
Length of hospital stay (days)		35.83 ± 33.06	19.71 ± 19.00	<0.001

A comparative analysis of the clinical characteristics between the IPN (*n* = 101) and NPN (*n* = 214) groups revealed no significant disparities with respect to sex, diabetes, hypertension, hyperlipidemia, and coronary heart disease. However, patients in the IPN cohort were notably older (*p* = 0.038), exhibited a more significant requirement for mechanical ventilation (*p* < 0.001), and experienced significantly prolonged hospital admissions (*p* < 0.001) compared to their NPN counterparts. While a higher incidence of hypoxemia was observed among IPN patients, this discrepancy did not reach statistical significance. These observations proffer critical insights for subsequent inquiries into the pathophysiology and prognostic determinants of IPN.

### 3.2 Segmentation results

**Figure 5:**
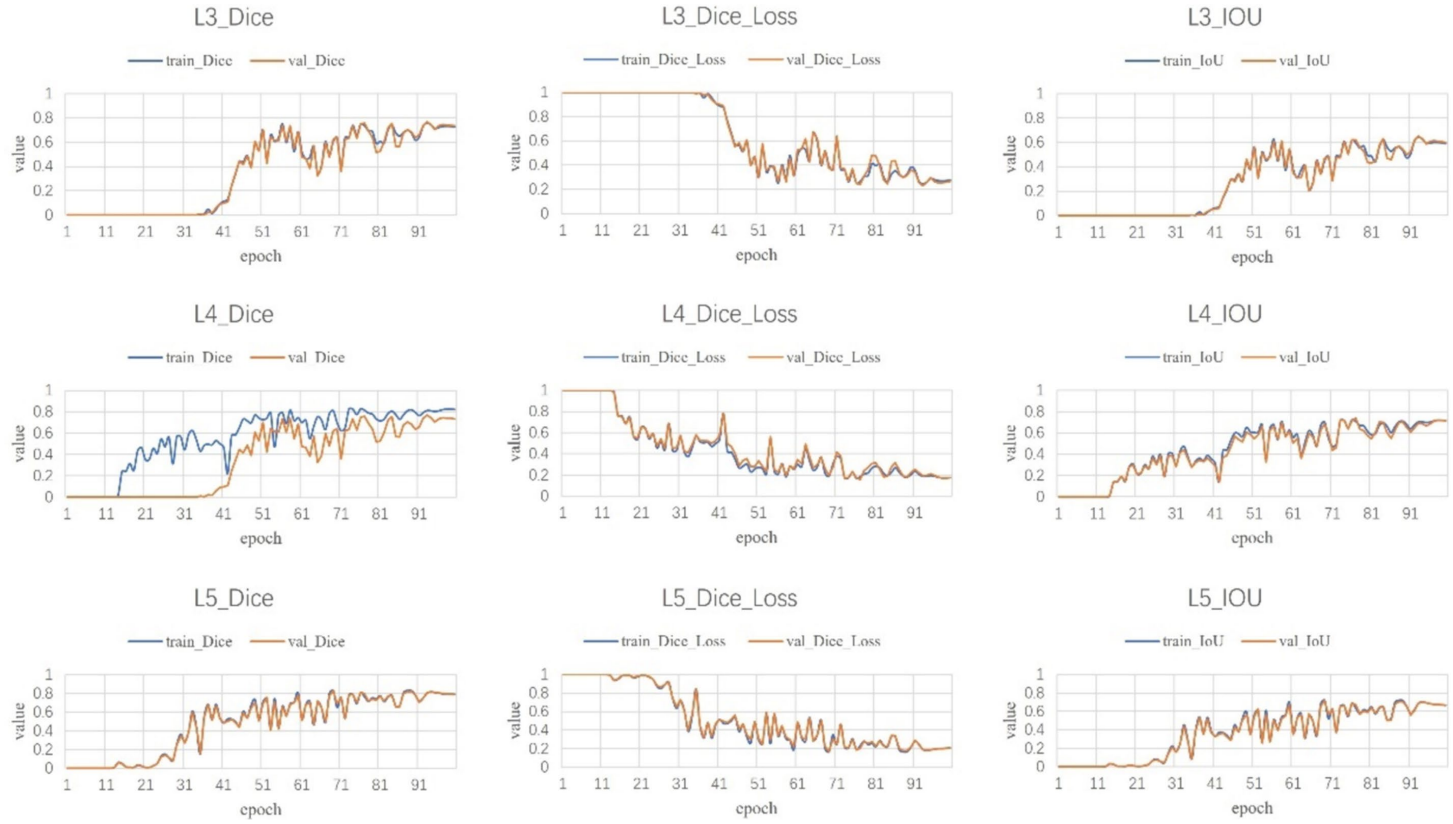
L3–L5 segmentation results.

**Figure 6:**
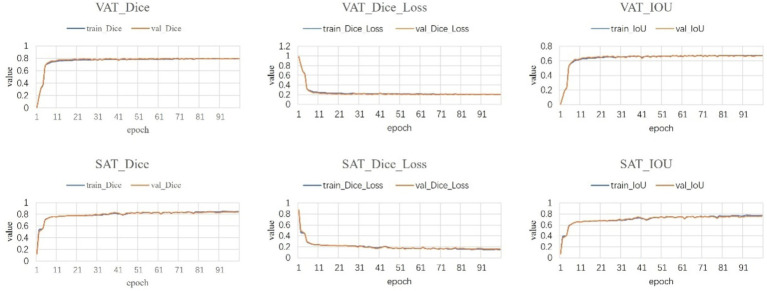
Fat segmentation results.

**Figure 7:**
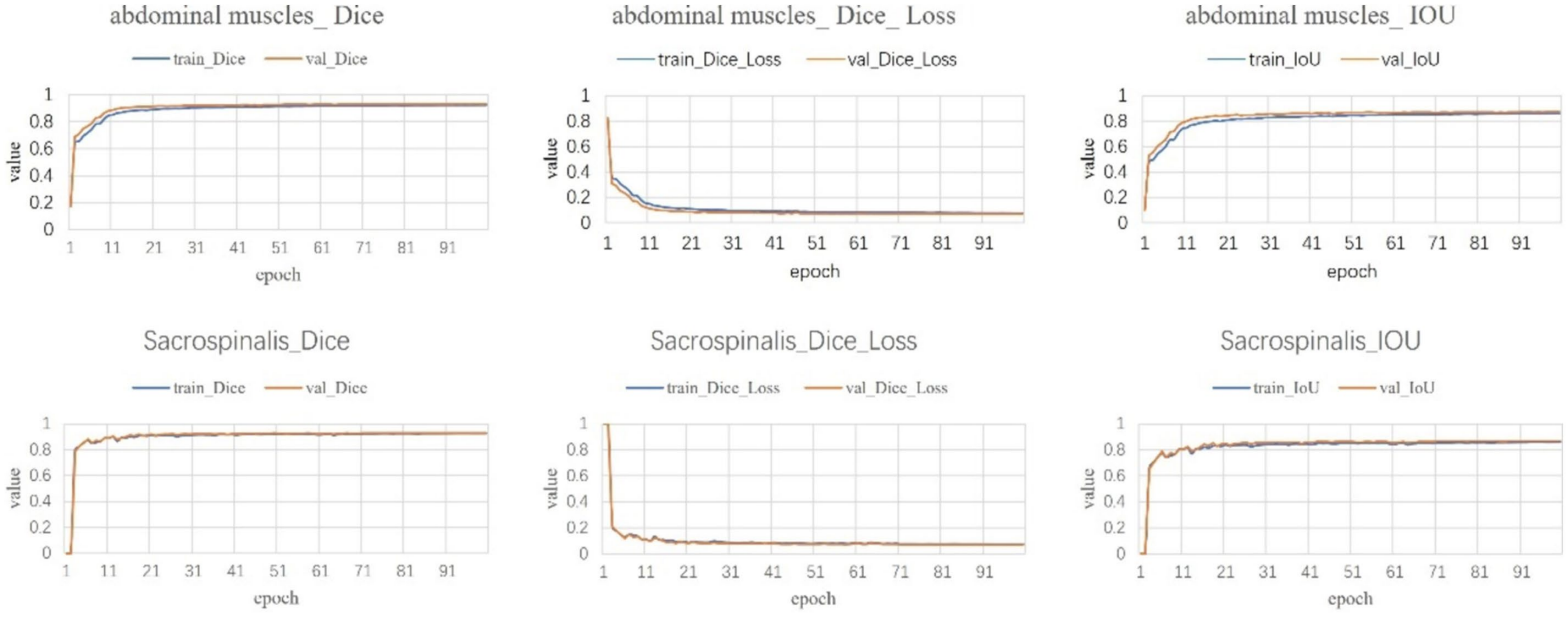
Muscle segmentation results.

Following a comprehensive assessment across five validation datasets, utilizing a 5-fold cross-validation approach with each model undergoing 100 training epochs, the model exhibiting superior performance was designated as the definitive model (Figures 5–7). The optimal model achieved an accuracy of 0.91 in segmenting the L3–L5 vertebral region within the validation dataset.

**Figure 8:**
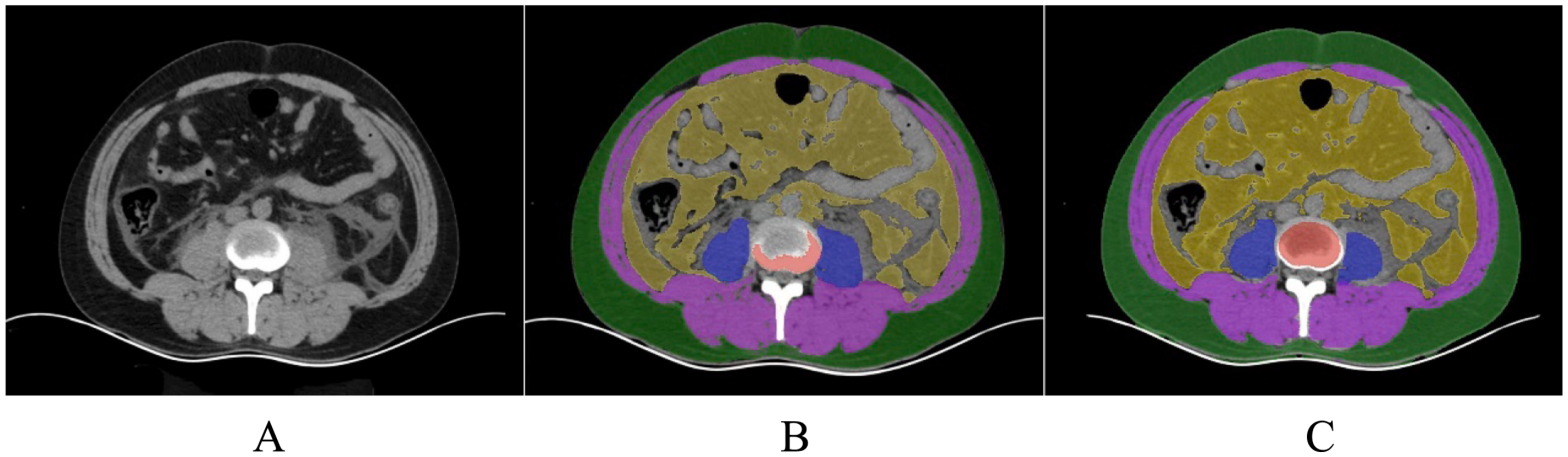
**(A)** Original image of a patient with pancreatitis. **(B)** A senior radiologist with over 15 years of experience manually delineates the ROI for assessing the effectiveness of segmenting human body components in patients with pancreatitis under human visualization (using a model not trained for pancreatitis). **(C)** Model-segmented ROI.

Within the L3–L5 vertebral range, we executed segmentation of the SAT, VAT, sacrospinalis, and all abdominal musculature (Figure 8).

### 3.3 Correlation between body composition and infectious pancreatic necrosis

**Figure 9:**
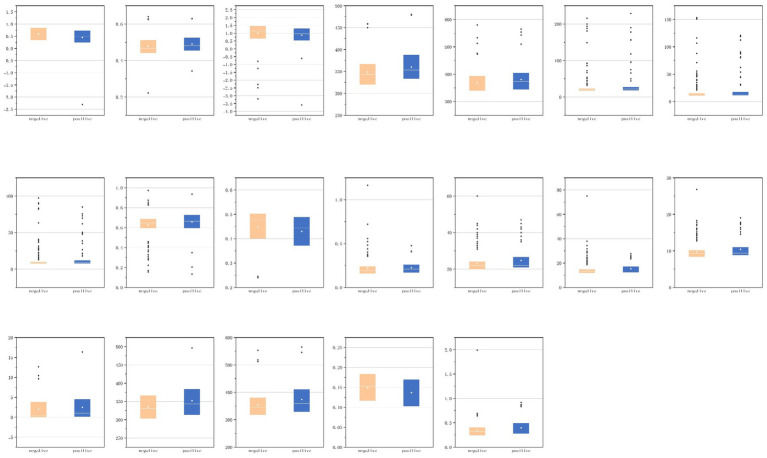
Radiomic features with statistical significance in body composition.

We conducted a detailed analysis of the first-order and shape characteristics of the segmented VAT, SAT, sacrospinalis, and all abdominal muscles. For the statistically significant features, we attempted to provide explanations and presented them in the form of box plots (Figure 9).

#### 3.3.1 VAT

As shown in Table 3, for the first-order VAT features, skewness (*p* = 0.004) and uniformity (*p* = 0.036) were statistically significant; the other features were not.

**Table 3:** Correlation between visceral adipose tissue (VAT) radiomic features and infectious pancreatic necrosis (IPN).

Feature	Negative	Positive	Statistical methods	Statistic	*p*
10 Percentile	−113.00 (−118.00, −107.00)	−112.27	Mann–Whitney U	9057.5	0.087
90 Percentile	−50.00 (−58.00, −44.00)	−50.75	Mann–Whitney U	9560.5	0.308
Energy	5559141605.50 (3540920150.50, 7802701555.75)	5517645106.00 (3029784775.75, 8244608979.00)	Mann–Whitney U	10,517	0.766
Entropy	1.26 (1.19, 1.32)	1.23 (1.17, 1.30)	Mann–Whitney U	11,721	0.05
IQR	31.00 (28.00, 35.00)	31.73	Mann–Whitney U	10,240	0.935
Kurtosis	3.05 (2.65, 3.72)	2.85 (2.55, 3.43)	Mann–Whitney U	11,718	0.051
Maximum	30.00 (16.00, 41.75)	21.50 (12.00, 41.25)	Mann–Whitney U	11,696.5	0.054
Mean	−84.93	−84.93	*t*-test	1.78111074	0.076
MAD	18.87 (17.33, 20.14)	18.47 (17.20, 20.04)	Mann–Whitney U	10,971	0.356
Median	−89.00 (−96.00, −82.00)	−88.61	Mann–Whitney U	8,889	0.052
Minimum	−233.00 (−301.50, −187.25)	−220.50 (−282.00, −175.00)	Mann–Whitney U	9,205	0.132
Range	268.00 (219.00, 337.25)	247.50 (202.75, 317.75)	Mann–Whitney U	11,636	0.066
Robust MAD	13.40 (11.99, 14.55)	13.24 (11.79, 14.80)	Mann–Whitney U	10,482	0.803
Root mean squared	88.78 (82.43, 94.47)	88.32	Mann–Whitney U	11,603	0.073
Skewness	0.6	0.48 (0.26, 0.72)	Mann-–Whitney U	12,390	0.004
Total energy	12114180613.50 (8090181675.25, 17111404744.75)	12041332397.50 (6156355601.50, 18040494741.50)	Mann–Whitney U	10,499	0.785
Uniformity	0.47 (0.44, 0.51)	0.48 (0.46, 0.52)	Mann–Whitney U	8,779	0.036
Variance	556.66 (488.00, 616.44)	527.72 (467.94, 606.80)	Mann–Whitney U	11,270	0.182
Elongation	0.61 (0.54, 0.68)	0.61 (0.56, 0.67)	Mann–Whitney U	9,754	0.452
Flatness	0.40 (0.35, 0.45)	0.40 (0.36, 0.47)	Mann–Whitney U	9,526	0.287
Least axis length	110.84 (104.00, 121.47)	111.99 (105.00, 140.59)	Mann–Whitney U	9,345	0.189
Major axis length	285.63 (264.87, 311.07)	291.49 (277.58, 313.18)	Mann–Whitney U	9,020	0.078
Maximum 2D diameter column	288.81 (268.45, 308.03)	292.30 (275.25, 314.29)	Mann–Whitney U	9,355	0.193
Maximum 2D diameter row	205.30 (182.48, 229.82)	208.38 (187.62, 233.90)	Mann–Whitney U	9,794	0.486
Maximum 2D diameter slice	276.66 (257.45, 300.00)	284.43 (267.16, 301.85)	Mann–Whitney U	9,070	0.09
Maximum 3D diameter	295.28 (272.07, 315.35)	296.97 (279.89, 326.37)	Mann–Whitney U	9,335	0.184
Mesh volume	1594888.51 (1116303.11, 2059782.88)	1565850.12 (994337.63, 2122169.49)	Mann–Whitney U	10,245	0.94
Minor axis length	176.57 (154.86, 198.44)	177.83 (157.36, 205.76)	Mann–Whitney U	9,538	0.294
Sphericity	0.19 (0.16, 0.21)	0.18	Mann–Whitney U	11,307	0.166
Surface area	348265.70 (276579.17, 415143.35)	345724.42 (280669.46, 450891.29)	Mann–Whitney U	9,911	0.593
Surface volume ratio	0.23 (0.19, 0.29)	0.24 (0.20, 0.31)	Mann–Whitney U	9,719	0.424
Voxel volume	1595786.56 (1117477.57, 2062007.94)	1577308.92 (998146.19, 2124513.29)	Mann–Whitney U	10,240	0.935

The skewness feature of VAT is correlated with the symmetry of VAT pixel distribution, with higher skewness values indicating excessive fat accumulation in certain areas and relatively less in others; within this set of features, skewness is 0.60 in the negative group, and lower in the positive group (0.48), suggesting that VAT in IPN patients exhibits less localized accumulation.

The uniformity feature pertains to whether VAT is evenly distributed within the ROI, with values closer to 1 indicating a more uniform texture and values farther from 1 suggesting greater heterogeneity. The negative group exhibits slightly lower uniformity than the positive group (0.47 compared to 0.48), indicating that the VAT density in IPN patients is more uniform.

#### 3.3.2 SAT

As shown in Table 4, for the first-order features of SAT, skewness (*p* = 0.023) showed statistical significance; among the shape features, maximum two-dimensional (2D) diameter slice (*p* = 0.020) and maximum three-dimensional (3D) diameter (*p* = 0.044) were statistically significant; the other features were not.

**Table 4:** Correlation between subcutaneous adipose tissue (SAT) radiomic features and infectious pancreatic necrosis (IPN).

Feature	Negative	Positive	Statistical methods	Statistic	*p*
10 Percentile	−116.00 (−121.00, −111.00)	−116.00 (−120.00, −110.00)	Mann–Whitney U	9,656	0.453
90 Percentile	−61.00 (−72.00, −52.00)	−60.59	Mann–Whitney U	9,035	0.107
Energy	7382051504.50 (4423188997.75, 10268867202.75)	7420446548.00 (4086489735.00, 10569815384.00)	Mann–Whitney U	10,304	0.883
Entropy	1.23 (1.13, 1.32)	1.23	Mann–Whitney U	9,958	0.741
IQR	25.00 (21.00, 31.00)	27.00 (22.00, 31.50)	Mann-–Whitney U	8,953.5	0.084
Kurtosis	5.48 (4.11, 7.55)	5.12 (3.45, 6.55)	Mann–Whitney U	11,508	0.069
Maximum	57.50 (35.25, 125.75)	48.00 (20.00, 104.00)	Mann–Whitney U	11,139	0.192
Mean	−93.04 (−98.62, −87.12)	−92.63 (−97.19, −82.42)	Mann–Whitney U	9,097	0.127
MAD	17.07 (14.82, 19.31)	17.39	Mann–Whitney U	9,674	0.469
Median	−98.00 (−103.75, −92.25)	−97.00 (−101.00, −87.00)	Mann–Whitney U	8,987.5	0.093
Minimum	−301.00 (−416.00, −195.50)	−296.00 (−383.00, −203.50)	Mann–Whitney U	9,966.5	0.75
Range	392.50 (243.25, 536.25)	350.00 (237.50, 511.00)	Mann–Whitney U	10,705	0.482
Robust MAD	10.93 (9.08, 13.27)	11.4	Mann–Whitney U	9,067	0.117
Root mean squared	95.87 (90.82, 100.83)	95.92 (86.10, 99.67)	Mann–Whitney U	11,329	0.117
Skewness	1.14 (0.67, 1.44)	0.96 (0.55, 1.27)	Mann–Whitney U	11,835	0.023
Total energy	15276971006.50 (10189081819.00, 22145540431.25)	15458624133.00 (10131235701.00, 22930332683.00)	Mann–Whitney U	10,199	0.998
Uniformity	0.47 (0.45, 0.52)	0.47 (0.44, 0.50)	Mann–Whitney U	10,760	0.435
Variance	508.17 (410.41, 646.90)	516.61 (397.99, 650.74)	Mann–Whitney U	10,310	0.876
Elongation	0.78 (0.73, 0.83)	0.79 (0.72, 0.84)	Mann–Whitney U	10,009	0.795
Flatness	0.26 (0.24, 0.30)	0.27 (0.24, 0.34)	Mann–Whitney U	9,773	0.557
Least axis length	112.77 (105.52, 124.29)	113.79 (105.34, 146.02)	Mann–Whitney U	9,525	0.352
Major axis length	433.34 (402.80, 460.00)	434.86	Mann–Whitney U	9,442	0.295
Maximum 2D diameter column	350.19 (334.44, 383.90)	362.97 (338.26, 397.18)	Mann–Whitney U	8,978	0.091
Maximum 2D diameter row	283.24 (263.04, 305.95)	286.41 (261.52, 314.55)	Mann–Whitney U	9,870	0.651
Maximum 2D diameter slice	342.91 (320.82, 366.55)	353.30 (334.67, 385.09)	Mann–Whitney U	8,521	0.02
Maximum 3D diameter	358.72 (340.51, 392.78)	374.06 (345.72, 403.56)	Mann–Whitney U	8,742	0.044
Mesh volume	1653557.96 (1235842.07, 2195203.36)	1680448.40 (1204551.02, 2591183.65)	Mann–Whitney U	9,960	0.743
Minor axis length	337.98 (312.61, 373.83)	338.29	Mann–Whitney U	9,658	0.455
Sphericity	0.25	0.25	*t*-test	1.448543564	0.148
Surface area	255759.30 (220992.29, 311631.51)	267878.28 (219325.30, 333155.36)	Mann–Whitney U	9,592	0.402
Surface volume ratio	0.16 (0.13, 0.21)	0.17 (0.12, 0.22)	Mann–Whitney U	9,590	0.4
Voxel volume	1654703.97 (1238486.14, 2195450.68)	1680171.98 (1205763.96, 2590036.86)	Mann–Whitney U	9,973	0.757

IQR, interquartile range; MAD, mean absolute deviation.

The skewness feature of SAT is higher in the negative group (1.14) compared to the positive group (0.96), indicating that SAT in IPN patients has less localized accumulation.

The maximum 2D diameter slice measures the maximum diameter of the SAT area in a 2D image, reflecting the extent of SAT expansion in the axial plane; the average maximum 2D diameter slice in the positive group (362.97) is greater than that in the negative group (350.19). The maximum 3D diameter reflects the overall size and shape of SAT in 3D space. Similarly, the maximum 3D diameter in the positive group (374.06) is larger than that in the negative group (358.72). Both features suggest that the volume of subcutaneous adipose tissue in IPN patients is larger relative to NPN patients.

#### 3.3.3 Abdominal muscles

As shown in Table 5, for the abdominal muscles’ first-order features, interquartile range (*p* = 0.023), mean absolute deviation (*p* = 0.039), and robust mean absolute deviation (*p* = 0.015) were statistically significant; among the shape features, elongation (*p* = 0.025), sphericity (*p* = 0.010), and surface volume ratio (*p* = 0.014) were statistically significant; and the other features were not.

**Table 5:** Correlation between abdominal muscles radiomic features and infectious pancreatic necrosis (IPN).

Feature	Negative	Positive	Statistical methods	Statistic	*p*
10 Percentile	30.00 (25.00, 35.00)	28.50 (21.00, 35.00)	Mann–Whitney U	11,216	0.207
90 Percentile	69.00 (64.00, 75.00)	69.00 (62.00, 78.00)	Mann–Whitney U	10,101.5	0.785
Energy	239309670.50 (155707966.75, 357551615.00)	241836573.50 (122022174.50, 368706009.75)	Mann–Whitney U	10,375	0.918
Entropy	1.04 (0.99, 1.09)	1.04 (0.99, 1.15)	Mann–Whitney U	9,891	0.574
IQR	20.00 (18.00, 23.00)	22.00 (19.00, 27.00)	Mann–Whitney U	8,649	0.023
Kurtosis	4.55 (3.81, 8.97)	4.80 (3.84, 9.29)	Mann–Whitney U	10,139	0.825
Maximum	147.50 (115.00, 351.75)	171.00 (119.75, 510.00)	Mann–Whitney U	9,185	0.125
Mean	49.46 (44.72, 54.10)	50.00 (42.57, 54.91)	Mann–Whitney U	10,608	0.672
MAD	12.64 (11.20, 14.75)	13.21 (11.66, 17.42)	Mann–Whitney U	8,799	0.039
Median	50.00 (45.25, 54.00)	50.50 (43.00, 55.00)	Mann–Whitney U	10,501.5	0.782
Minimum	−63.50 (−75.00, −50.00)	−64.00 (−82.50, −51.50)	Mann–Whitney U	10,818	0.476
Range	213.00 (179.25, 429.00)	247.50 (184.75, 672.50)	Mann–Whitney U	9,092	0.096
Robust MAD	8.58 (7.67, 9.77)	9.19 (8.12, 11.61)	Mann–Whitney U	8,527	0.015
Root mean squared	52.48 (47.75, 56.90)	53.14 (45.53, 58.53)	Mann–Whitney U	10,322	0.976
Skewness	−0.29 (−0.51, 0.31)	−0.23 (−0.47, 0.72)	Mann–Whitney U	9,782	0.476
Total energy	504705905.60 (331904476.82, 745238186.75)	505832574.90 (277667575.85, 814704451.82)	Mann–Whitney U	10,407	0.883
Uniformity	0.51 (0.49, 0.54)	0.50 (0.49, 0.55)	Mann–Whitney U	10,606	0.674
Variance	274.30 (214.07, 396.96)	295.51 (227.77, 519.59)	Mann–Whitney U	8,972	0.067
Elongation	0.64 (0.60, 0.69)	0.67 (0.60, 0.72)	Mann–Whitney U	8,669	0.025
Flatness	0.23 (0.20, 0.26)	0.23 (0.21, 0.28)	Mann–Whitney U	9,539	0.295
Least axis length	43.31 (36.50, 49.07)	42.02 (36.22, 51.25)	Mann–Whitney U	10,513	0.77
Major axis length	178.21 (166.32, 199.38)	175.26 (166.77, 199.52)	Mann–-Whitney U	10,828	0.467
Maximum 2D diameter column	149.23 (140.63, 186.94)	147.74 (138.45, 201.81)	Mann–Whitney U	10,701	0.581
Maximum 2D diameter row	117.22 (109.44, 134.28)	118.61 (107.65, 148.15)	Mann–Whitney U	9,896	0.578
Maximum 2D diameter Slice	140.85 (132.95, 159.86)	139.52 (131.26, 163.66)	Mann–Whitney U	10,818	0.476
Maximum 3D diameter	163.09 (153.48, 222.48)	161.51 (152.06, 226.11)	Mann–Whitney U	10,598	0.682
Mesh volume	170053.90 (124849.08, 226962.00)	158223.70 (114210.53, 203752.58)	Mann–Whitney U	11,066	0.292
Minor axis length	114.68 (107.51, 125.67)	115.75 (105.68, 144.08)	Mann–Whitney U	9,822	0.511
Sphericity	0.48 (0.40, 0.50)	0.44 (0.37, 0.49)	Mann–Whitney U	12,166	0.01
Surface area	32950.71 (26557.99, 38100.90)	32240.91 (25502.50, 39481.43)	Mann–Whitney U	10,503	0.78
Surface volume ratio	0.19 (0.16, 0.24)	0.21 (0.17, 0.26)	Mann–Whitney U	8,516	0.014
Voxel volume	170378.76 (125078.19, 227293.64)	158435.95 (114481.55, 203957.85)	Mann–Whitney U	11,068	0.29

IQR, interquartile range; MAD, mean absolute deviation.

The interquartile range (IQR) of the abdominal muscles reflects their stability or variability under different conditions, describing the degree of dispersion in data distribution. It represents the range of the middle 50% of the data. The IQR in the negative group is less than that in the positive group (20.00 vs. 22.00). This may indicate that in patients who develop infectious pancreatic necrosis, the signal intensity distribution of the abdominal muscles is more dispersed.

Mean absolute deviation (MAD) is another statistical measure of data distribution dispersion, which quantifies the average distance of data points from the mean. The robust mean absolute deviation is a more robust version of MAD, insensitive to outliers. The findings of these two features are consistent, indicating that the signal intensity distribution of the abdominal muscles in patients who develop infectious pancreatic necrosis may be more uneven.

Elongation reflects the extent of longitudinal extension of muscle fibers or muscle blocks, with the elongation rate in the positive group being greater than that in the negative group (0.67 vs. 0.64). This may suggest that the shape of the abdominal muscles in patients who develop infectious pancreatic necrosis is more elongated in one direction.

Sphericity measures the similarity of the shape of an ideal sphere, reflecting the compactness and regularity of the shape of muscle fibers or muscle blocks; the sphericity in the negative group is greater than that in the positive group (0.48 vs. 0.44). This may imply that the shape of the abdominal muscles in patients who do not develop infectious pancreatic necrosis is closer to spherical. In contrast, the shape of the abdominal muscles in patients who develop infectious pancreatic necrosis may become less regular due to inflammation or other pathological changes.

The surface volume ratio reflects the ratio of muscle surface area to volume, with the positive group having a higher surface volume ratio than the negative group (0.21 vs. 0.19). This may indicate that the abdominal muscles of patients who develop infectious pancreatic necrosis have a relatively larger surface area in proportion to volume, possibly reflecting tissue edema due to the inflammatory response.

#### 3.3.4 Sacrospinalis

As shown in Table 6, for the first-order features of sacrospinalis, interquartile range (*p* = 0.018), mean absolute deviation (*p* = 0.049), robust mean absolute deviation (*p* = 0.025), and skewness (*p* = 0.008) exhibited statistical significance; among the shape features, maximum 2D diameter slice (*p* = 0.008), maximum 3D diameter (*p* = 0.005), sphericity (*p* = 0.011), and surface volume ratio (*p* = 0.005) were statistically significant; the other features were not.

**Table 6:** Correlation between sacrospinalis radiomic features and infectious pancreatic necrosis (IPN).

Feature	Negative	Positive	Statistical methods	Statistic	*p*
10 Percentile	21.00 (16.00, 25.00)	21.22	Mann–Whitney U	11,298	0.169
90 Percentile	63.00 (58.00, 69.00)	63.50 (57.75, 72.50)	Mann–Whitney U	9,998	0.678
Energy	1249152060.00 (764619051.75, 1994064418.25)	1187720175.50 (668420562.50, 2087340047.00)	Mann–Whitney U	10,681	0.6
Entropy	0.99 (0.86, 1.04)	1.00 (0.85, 1.09)	Mann–Whitney U	9,841	0.528
IQR	22.00 (20.00, 24.00)	22.00 (21.00, 26.25)	Mann–Whitney U	8,587.5	0.018
Kurtosis	10.42 (5.55, 61.15)	23.54 (5.97, 66.70)	Mann–Whitney U	9,273	0.157
Maximum	526.50 (382.25, 756.25)	528.50 (402.00, 796.25)	Mann–Whitney U	9,686.5	0.398
Mean	43.95 (38.19, 48.39)	43.25 (37.46, 49.67)	Mann–Whitney U	10,389	0.903
MAD	13.25 (12.16, 14.78)	13.39 (12.62, 16.81)	Mann–Whitney U	8,868	0.049
Median	45.00 (39.00, 49.00)	45.2	Mann–Whitney U	10,492.5	0.791
Minimum	−44.00 (−57.00, −34.00)	−44.00 (−58.25, −29.00)	Mann–Whitney U	10,068.5	0.75
Range	579.50 (415.50, 814.25)	571.50 (445.75, 847.75)	Mann–Whitney U	9,730.5	0.433
Robust MAD	9.29 (8.43, 10.13)	9.40 (8.92, 10.94)	Mann–Whitney U	8,671	0.025
Root mean squared	47.21 (41.66, 51.93)	47.15 (41.68, 55.19)	Mann–Whitney U	10,241	0.936
Skewness	0.32 (0.01, 3.77)	1.01 (0.19, 4.42)	Mann–Whitney U	8,375	0.008
Total energy	2847674195.50 (1720109062.25, 4205653583.50)	2575449699.50 (1406155363.00, 4910820334.25)	Mann–Whitney U	10,661	0.619
Uniformity	0.54 (0.50, 0.63)	0.54 (0.50, 0.63)	Mann–Whitney U	10,773	0.515
Variance	286.91 (244.39, 468.43)	307.96 (256.65, 654.50)	Mann–Whitney U	9,045	0.084
Elongation	0.68	0.68	*t*-Test	1.31513754	0.189
Flatness	0.32 (0.29, 0.37)	0.32 (0.29, 0.42)	Mann–Whitney U	10,156	0.843
Least axis length	108.65 (101.74, 120.38)	109.74 (102.19, 151.06)	Mann–Whitney U	9,466	0.251
Major axis length	346.65 (320.52, 374.80)	356.08 (327.14, 381.18)	Mann–Whitney U	8,989	0.071
Maximum 2D diameter column	331.83 (307.16, 355.28)	334.92 (309.67, 366.12)	Mann–Whitney U	9,127	0.106
Maximum 2D diameter row	263.36 (238.13, 296.13)	265.74 (231.65, 304.67)	Mann–Whitney U	10,007	0.687
Maximum 2D diameter slice	330.38 (303.91, 365.72)	342.84 (313.74, 382.86)	Mann–Whitney U	8,369.5	0.008
Maximum 3D diameter	343.67 (318.46, 379.83)	359.58 (329.37, 408.87)	Mann–Whitney U	8,284	0.005
Mesh volume	1211160.13 (843465.21, 1518283.12)	1119268.25 (755711.11, 1583688.32)	Mann–Whitney U	10,954	0.368
Minor axis length	234.46 (212.14, 258.54)	238.73 (205.35, 263.39)	Mann–Whitney U	9,996	0.676
Sphericity	0.15 (0.12, 0.18)	0.15	Mann–Whitney U	12,137	0.011
Surface area	324534.33 (273229.10, 450328.40)	338175.63 (268258.35, 528150.56)	Mann–Whitney U	9,860	0.545
Surface volume ratio	0.32 (0.25, 0.40)	0.35 (0.28, 0.49)	Mann–Whitney U	8,247	0.005
Voxel volume	1211242.46 (846322.89, 1519310.32)	1117691.58 (755909.58, 1586327.08)	Mann–Whitney U	10,966	0.359

IQR, interquartile range; MAD, mean absolute deviation.

The interquartile range of the sacrospinalis is equal in the median for both groups, but the confidence interval for the positive group is greater than that for the negative group; the results of MAD and robust MAD are consistent with those of the abdominal muscles, indicating a more uneven signal intensity distribution in the IPN group; the median Sphericity is equal for both groups, but the confidence interval for the positive group is smaller than that for the negative group; the surface volume ratio for the positive group is greater than that for the negative group (0.35 vs. 0.32). These findings are consistent with the overall abdominal muscles.

The skewness feature of the sacrospinalis shows statistical significance, whereas there is no statistical difference in the abdominal muscles, with the median of the positive group being greater than that of the negative group (1.01 vs. 0.32), indicating that the intensity distribution of the sacrospinalis in IPN patients may be more uneven.

For the sacrospinalis, the maximum 2D diameter slice and maximum 3D diameter exhibit statistical significance, whereas there is no statistical difference for the abdominal muscles. These features have significant discriminative or representative value in assessing the morphology, structure, or function of the sacrospinalis. Both features are greater in the positive group than the negative group, indicating that the volume of the sacrospinalis in IPN patients is larger in NPF patients.

## 4 Discussion

IPN, as a severe complication of ANP, poses a significant clinical challenge due to its high mortality rate and incidence (Li et al., 2022). However, prolonged antibiotic use in the absence of infection may lead to multidrug-resistant bacterial infections, further increasing mortality (Lu et al., 2022). Therefore, early and accurate diagnosis of IPN followed by timely and effective treatment measures is crucial. This study delineated muscles and fat tissues using deep learning techniques based on non-contrast CT images. Subsequently, we extracted 18 first-order features and 14 shape features using radiomic techniques and conducted a correlation analysis. Notably, we provided detailed medical interpretations for the statistically significant features, revealing the potential physiological and pathological significance behind these features. This study highlights the important role of body composition, including muscles and fat tissues, in acute pancreatitis. It provides a quantitative evaluation tool based on highly standardized and readily available non-contrast CT data using artificial intelligence.

The application of deep learning enables precise segmentation of muscle and fat (Graffy et al., 2019; Shen et al., 2023), which is crucial for delving into the complexity of infectious diseases. It not only enhances our comprehension of the disease’s pathophysiological mechanisms but also significantly enriches the means of assessing patients’ nutritional status and inflammatory responses. For instance, the study by Zhang et al. explored radiomic features from CT images of 1,245 adrenal glands and surrounding fat tissues, strongly demonstrating their correlation with disease progression in COVID-19 patients (Zhang M. et al., 2023). Similarly, Yoo et al. utilized deep learning techniques to quantify liver and spleen volumes, as well as SAT and VAT tissues and skeletal muscle indices, providing valuable prognostic information for patients with chronic hepatitis B (CHB) (Yoo et al., 2023). Compared to traditional assessment methods (such as modified CT severity index [MCTSI], Ranson, bedside index for severity in acute pancreatitis [BISAP], etc.), these methods, while concise and easy to understand, have limitations due to the limited factors they consider, making it difficult to fully reflect the complexity and dynamic changes of diseases. Previous studies have primarily focused on the pancreas itself (Zhang C. et al., 2024; Xue et al., 2023; Lin et al., 2020; Chen et al., 2023). In our study, the detailed evaluation of VAT, SAT, abdominal muscles, and sacrospinalis not only deepened our understanding of the disease’s pathophysiological mechanisms but also significantly improved our ability to assess patients’ nutritional status and inflammatory responses. Through in-depth analysis of radiomic data, we found that some parameters showed a good correlation with disease states, further validating the reliability and effectiveness of these body composition indicators as disease assessment tools. This discovery not only emphasizes the importance of muscle and fat segmentation in the evaluation of infectious diseases but also lays a solid foundation for the future development of more precise and comprehensive disease assessment systems.

The World Health Organization defines obesity as a pathological condition characterized by excessive accumulation of body fat, with a body mass index (BMI) of ≥30 kg/m^2^ (Whitlock et al., 2009), which exerts certain effects on inflammation (Ponce-de-Leon et al., 2022). Numerous epidemiological studies and meta-analyses have demonstrated that obesity is a prognostic factor affecting the severity of acute pancreatitis (AP) (Chen et al., 2012; Martínez et al., 2006; Cruz-Monserrate et al., 2016; Wang et al., 2011). This study found significant correlations between certain radiomic features of SAT and VAT and the occurrence of IPN: our results suggest that for patients developing IPN, less localized accumulation of VAT and SAT, uniform density of VAT and SAT, and a larger volume of SAT are risk factors. These findings may be related to age and sex (Zhou et al., 2022; Lizcano and Guzmán, 2014; Tchernof and Després, 2013; Frank et al., 2019; Palmer and Kirkland, 2016; Pascot et al., 1999): under the influence of sex hormones, men tend to accumulate fat tissue predominantly in visceral regions, while women accumulate it more subcutaneously, leading to symmetrical differences in fat distribution between genders; with advancing age, the distribution of fat in both genders also changes, which may affect the localized accumulation and uniformity of VAT density, thereby explaining why adjusted body composition parameters based on age and sex in previous studies could more accurately predict the severity of AP and avoid the fat paradox (Horibe et al., 2022). Furthermore, we hypothesize that the statistical significance of skewness features may also be associated with the distribution of fat necrosis; if so, our study’s results suggest that the inflammatory or necrotic process is more diffused in the adipose tissue of IPN patients, or there may be more synchronized pathological changes. Our conclusions indicate that the volume of SAT has a positive effect on the occurrence of IPN, and we speculate that the increased volume of subcutaneous adipose tissue may be related to enhanced or systemic inflammatory responses, which could exacerbate the infection of necrotic pancreatic tissue.

Skeletal muscle, as a pivotal component of the human body, not only supports fundamental motor functions but is also closely associated with individual metabolic status, inflammatory responses, and long-term disease prognosis (Akturk et al., 2021; Picca and Calvani, 2021; Modesto et al., 2020). In this study, we conducted an in-depth analysis of the sacrospinalis muscle and all abdominal muscles, including the sacrospinalis. Patients with IPN exhibited scattered and heterogeneous muscle density distributions in the overall abdominal muscles and the sacrospinalis, a significant difference compared to the NPN population. This alteration was manifested in an increased surface area-to-volume ratio and a morphological deviation from the ideal spherical structure. These findings suggest that such changes in muscle tissue may represent an adaptive response to the pathological state of IPN, and we have posited several hypotheses: First, the dispersion and heterogeneity of muscle density might be related to the level of inflammation within IPN patients. Inflammatory responses could lead to alterations in the intramuscular environment, affecting muscle cell growth, metabolism, and extracellular matrix remodeling (Tu and Li, 2023); second, the increased volume of the sacrospinalis, particularly in IPN patients, may reflect compensatory changes in muscle tissue during disease progression. The increase in muscle volume might be an adaptation to the additional load imposed by inflammation and metabolic disturbances, and it may also represent an attempt by the body to maintain essential physiological functions. The significant differences in skewness features indicate that the muscle density distribution in IPN patients deviates from the normal range, and the increased asymmetry may be related to the damage and repair processes in muscle tissue. During the continuous self-repair of muscle tissue, structural and functional asymmetries may arise due to the influence of an inflammatory environment.

While this study has made certain progress in body composition assessment and IPN prediction based on non-contrast CT, there are still some limitations. First, this is a single-center study with a relatively limited sample size, which may limit the generalizability of the findings. Second, this study did not quantitatively assess the degree of pancreatic infection, which may affect the in-depth understanding of the mechanism of IPN occurrence. Additionally, this study did not provide a detailed analysis of patient prognosis and length of hospital stay; future studies can further explore the relationship between body composition and the prognosis of pancreatitis patients. Finally, due to the unclear boundaries of pancreatitis, this study did not segment pancreatic lesions, which may limit the in-depth exploration of the pathophysiological mechanisms of pancreatitis itself.

## 5 Conclusion

This study, utilizing deep learning techniques in conjunction with unenhanced CT imaging, has elucidated the close association between muscle and fat tissue and the progression of ANP to IPN, providing a novel tool for early warning and personalized treatment of IPN. The research identified that first-order features of fat (such as skewness, uniformity, etc.), first-order features of muscle, and shape features (such as interquartile range, sphericity, etc.) are all significantly correlated with IPN. In-depth analysis revealed that less localized accumulation of VAT and SAT, uniform density of VAT and SAT, larger volume of SAT, and the dispersion and heterogeneity of abdominal muscle density distribution are all risk factors for IPN. This not only confirms the pivotal role of body composition in the progression of ANP but also provides a scientific basis for the implementation of early preventive treatment in clinical practice for high-risk patients. The findings of this study offer important references and guidance for improving the overall prognosis of pancreatitis patients and optimizing clinical management strategies.

## Data Availability

The original contributions presented in the study are included in the article/supplementary material, further inquiries can be directed to the corresponding author.
